# Comparisons of Mouse Mesenchymal Stem Cells in Primary Adherent Culture of Compact Bone Fragments and Whole Bone Marrow

**DOI:** 10.1155/2015/708906

**Published:** 2015-03-03

**Authors:** Yiting Cai, Tianshu Liu, Fang Fang, Chengliang Xiong, Shiliang Shen

**Affiliations:** ^1^Family Planning Research Institute, Tongji Medical College, Huazhong University of Science and Technology, Wuhan 430030, China; ^2^Department of Thoracic Surgery, Renmin Hospital of Wuhan University, Wuhan 430060, China; ^3^Center for Reproductive Medicine, Tongji Medical College, Huazhong University of Science and Technology, Wuhan 430010, China; ^4^Zhong Shen Bioscience Inc., Biolake B3-1, 666 Gaoxin Road, East Lake Hi-Tech Zone, Wuhan 430000, China

## Abstract

The purification of mouse bone marrow mesenchymal stem cells (BMSCs) by using the standard method of whole bone marrow adherence to plastic still remains ineffective. An increasing number of studies have indicated compact bone as an alternative source of BMSCs. We isolated BMSCs from cultured compact bone fragments and investigated the proliferative capacity, surface immunophenotypes, and osteogenic and adipogenic differentiations of the cells after the first trypsinization. The fragment culture was based on the fact that BMSCs were assembled in compact bones. Thus, the procedure included flushing bone marrow out of bone cavity and culturing the fragments without any collagenase digestion. The cell yield from cultured fragments was slightly less than that from cultured bone marrow using the same bone quantity. However, the trypsinized cells from cultured fragments exhibited significantly higher proliferation and were accompanied with more CD90 and CD44 expressions and less CD45 expression. The osteogenic and adipogenic differentiation capacity of cells from cultured fragments were better than those of cells from bone marrow. The directly adherent culture of compact bone is suitable for mouse BMSC isolation, and more BMSCs with potentially improved proliferation capacity can be obtained in the primary culture.

## 1. Introduction

Mesenchymal stem cells (MSCs) are intriguing adult stem cells with great potential in autoimmune disease treatment, tissue regeneration, and myocardial infarction therapy [1–6]. Friedenstein et al. [7] isolated bone marrow MSCs (BMSCs) using the adherent culture of whole bone marrow to plastic flask. In contrast to acquiring the BMSCs of humans, rats, rabbits, and some other species, acquiring abundant and homogeneous BMSCs from murine bone marrow by using the adherent culture is difficult [8–11]. Approximately 1 MSC per 10^6^ mononuclear cells is observed within the bone marrow of mice, thus indicating an exceedingly low frequency of MSCs in bone marrow. The culture expansion of homogeneous BMSCs relies on serial generations and prolonged culture, which lead to cell senescence and property variations [12].

According to studies from several independent research teams demonstrating that BMSCs live in compact bones, BMSCs that are derived from compact bones can be obtained by the adherent culture of collagenase-digested bone fragments after removing the released cells or by collagenase-released cell culture [13, 14]. The cells exhibit homogeneous surface antigen profile and tridifferentiation along osteocyte, chondrocyte, and adipocyte lineages as BMSCs from bone marrow. However, the possibility of BMSCs migrating from compact bones without any enzyme pretreatments is not well known. In this study, we isolated the BMSCs by using adherent culture of compact bone fragments without the pretreatment of collagenase and observed the yield, proliferative capacity, surface immunophenotypes, and osteogenic and adipogenic differentiations of cells of the first passage.

## 2. Material and Methods

All animal procedures were approved by the Ethical Committee of Huazhong Science and Technology University ([2013] IACUC number 337).

### 2.1. Isolation of Primary BMSCs

Four- to five-week-old male inbred BALB/c mice were purchased from the Laboratory Animal Center of Tongji College, Huazhong University of Science and Technology (Wuhan, China). The femurs and tibiae cleared of connective tissue and epiphyses were collected from the sacrificed mice. To quantify cells per one mouse obtained in primary culture, two femurs and two tibiae were used in each method as follows: (1) adherent culture of whole bone marrow and (2) adherent culture of bone fragments. The culture was kept in 5% CO_2_ incubator at 37°C for 48 h, and nonadherent cells were removed by phosphate-buffered saline (PBS). Thereafter, the medium was changed every three days. Once the cell confluence in either culture group reached 80%, the cells from cultured bone marrow and fragments were both trypsinized by 0.25% trypsin for 4 min and prepared as cells of the first passage for all the following analyses. The BMSC isolation experiment was repeated 6 times (*n* = 6).

#### 2.1.1. Adherent Culture of Whole Bone Marrow

The standard isolation aimed to flush BMSCs from the hind legs [7]. The whole marrow was flushed out of the bone cavities thoroughly by drawing and expelling with 1 mL syringe using PBS for 10 times. Marrow aspiration was filtrated by a 70 *μ*m nylon mesh filter, centrifuged, and plated into 3 wells of 6-well plates with 2 mL Dulbecco Modified Eagle Medium/F12 (DMEM/F12; HyClone, USA) supplemented with 15% fetal bovine serum (FBS; Gibco, USA), 100 U/mL penicillin, and 100 *μ*g/mL streptomycin (HyClone).

#### 2.1.2. Adherent Culture of Bone Fragments

After flushing the whole marrow out of bone cavities twice by PBS, the bones were dissected into fragments of 0.3 cm × 0.3 cm and were placed into 3 wells of 6-well plates (approximately 9 to 10 fragments per well). The fragments were wetted with 200 *μ*L medium overnight and with 1.5 mL medium on the next day to avoid fragments floating. The fragments were discarded on the fifth day. The adherent cells were then supplemented with fresh culture medium every three days.

### 2.2. Proliferative Analysis

#### 2.2.1. MTT (3-(4,5-Dimethylthiazol-2-yl)-2,5-diphenyltetrazolium Bromide) Assay for Growth Curve

Cells of the first passage were plated into 96-well plates at a density of 7,000 cells/well. On day 1 to day 6 after plating, 10 *μ*L 5 mg/mL MTT (Sigma, USA) was added into the incubation for 4 h. The absorbance was read at 490 nm by microplate reader (Awareness Tech. Inc., USA). Each sample was analyzed in triplicate.

#### 2.2.2. Colony-Forming Unit-Fibroblast (CFU-F) Assay

Cells of the first passage were plated into 60 mm dishes at densities of 800, 5,000, 10,000, and 20,000 cells/well for 14 days in a humidified condition of 5% CO_2_ at 37°C. The culture medium was changed on day 7, and the cells were fixed in 4% paraformaldehyde and stained with crystal violet (Sigma) on day 14. Colonies that displayed more than 5 cells were scored under an inverted microscope (Olympus, Japan). Each sample was analyzed in triplicate.

### 2.3. Flow Cytometry Analysis for the Immunophenotypes

Cells of the first passage were washed by PBS twice and stained with the respective antibodies directly conjugated to fluorescein isothiocyanate (FITC), including anti-mouse FITC-CD90, FITC-CD44, FITC-CD45, and FITC-CD34 (eBioscience, USA) at 4°C for 30 min. The corresponding mouse FITC-IgG2a was served as the isotype-matched control. The cell pellets were resuspended in 300 *μ*L PBS and examined with flow cytometry (Becton Dickinson, USA).

### 2.4. Osteogenic Differentiation Assay

Osteogenic differentiation was induced in a specific medium [15]. Cells of the first passage were seeded at a density of 50,000 cells/well in 24-well plates. The osteoinductive medium was changed every three days. The completion of the 3-week induction was assessed by alizarin red staining.

### 2.5. Adipogenic Differentiation Assay

To identify the adipogenic capacity, cells of the first passage were maintained in an adipoinductive medium containing 10^−6^ M dexamethasone and 10 *μ*g/mL insulin [15]. Lipid droplets were stained by oil red after the 2-week induction.

### 2.6. Statistical Analysis

Data were reported as median values (±SD) and analyzed with the Mann-Whitney test using PASW Statistics18 (IBM Deutschland GmbH, Germany). *P* < 0.05 was served as the statistical significance.

## 3. Results

### 3.1. Cell Yield and Morphological Analyses

**Figure 1:**
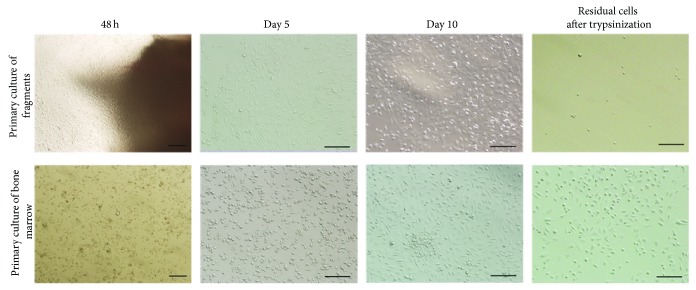
Morphology of primary cells from cultured bone marrow and bone fragments on days 2, 5, and 10 after plating. The residual cells adherent to plastic were observed after the trypsinization on day 10. Bar 100 *μ*m.

The cell yield of the first passage from cultured fragments was slightly less than that from cultured bone marrow using the same bone quantity (Table 1, *P* ≥ 0.05).  Figure 1 illustrated that cells crept from the fragments and adhered to the plastic after 48 h. On day 5, all fragments were discarded and approximately 80% of the adherent cells exhibited a spindle shape. Some colonies grew evidently after 10 days, at which time the confluence of the cells reached ~70%. In contrast to the cells cultured from fragments, the adherent cells from the bone marrow varied in obviously heterogeneous morphology, such as fusiform, irregular, and triangular cells on day 5. And the confluence reached ~80% after the 10-day culture. Compared with the residual cells after the trypsinization in cultured fragments, more spindle-shaped and irregular cells which remained adherent to the plastic in bone marrow cultivation were observed. Moreover, the cells could be serially subcultured for at least 6 generations, and presence of flattened cells was obvious after 10 generations, indicating BMSC senescence.

### 3.2. Potential of the Proliferation

**Figure 2:**
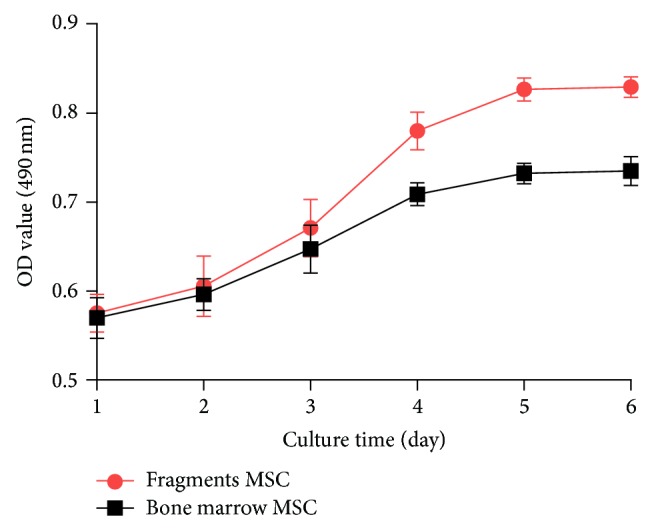
Cell growth curves of the first passage from cultured bone marrow and bone fragments. ^*^ *P* < 0.05, compared with bone marrow MSC at the same day.

Figure 2 illustrated that the cells of the first passage from cultured fragments and bone marrow showed a similar shape of “S” growth curve. However, the cells of cultured fragments obviously exhibited more rapid proliferation on day 4 to day 6 following a 2-day incubation compared with cells of cultured bone marrow on the respective day (*P* < 0.05).

**Figure 3:**
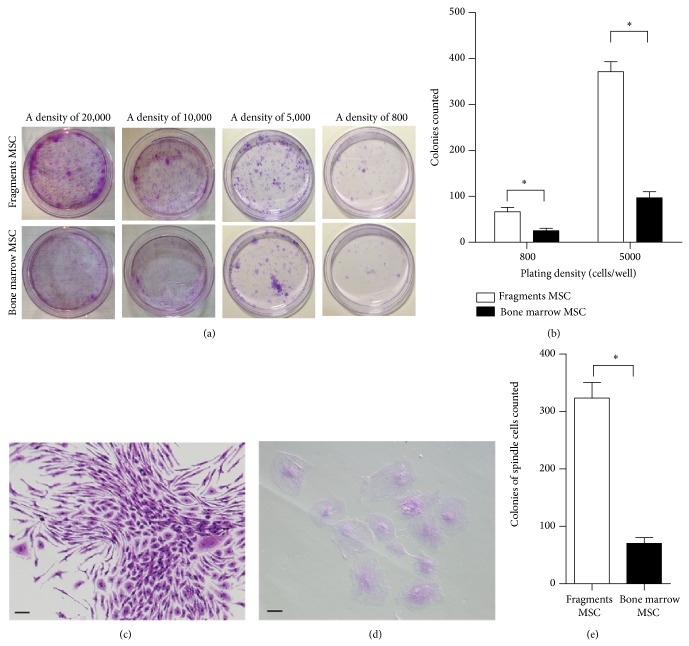
Analyses of cell CFU-F colonies of the first passage from cultured bone marrow and bone fragments. (a) Representative images of cell colonies at densities of 20,000, 10,000, 5,000, and 800 cells/well. (b) Colony counting analyses at densities of 5,000 and 800 cells/well. (c) Colony consisted of most of spindle cells. (d) Colony consisted of flattened cells. (e) Counting analyses for colonies which consisted of most of spindle cells at a density of 5,000 cells/well. Bar 20 *μ*m; ^*^ *P* < 0.05.

The CFU-F colonies of the cultured cells were further analyzed to assess the differential proliferation. Consistent with the MTT results, the proliferative capacity of the cells from cultured fragments improved compared with the cells from cultured bone marrow at high densities of 10,000 and 20,000 cells/well and low densities of 800 and 5,000 cells/well (Figure 3(a)). The colonies were quantified in Figure 3(b), which illustrated that the colonies of cultured fragments increased to approximately two times at a density of 800 cells/well and four times at a density of 5,000 cells/well compared with those of cultured bone marrow (*P* < 0.05). Furthermore, we observed two types of colony morphology. One was the colony which consisted mostly of the typically spindle cells, which were generally considered as real BMSCs (Figure 3(c)), and the other was the colony including the flattened cells (Figure 3(d)). The colony yield of spindle-shaped cells from cultured fragments was three times higher than that from cultured bone marrow at a density of 5,000 cells/well (Figure 3(e), *P* < 0.05).

### 3.3. Surface Immunophenotypes

**Figure 4:**
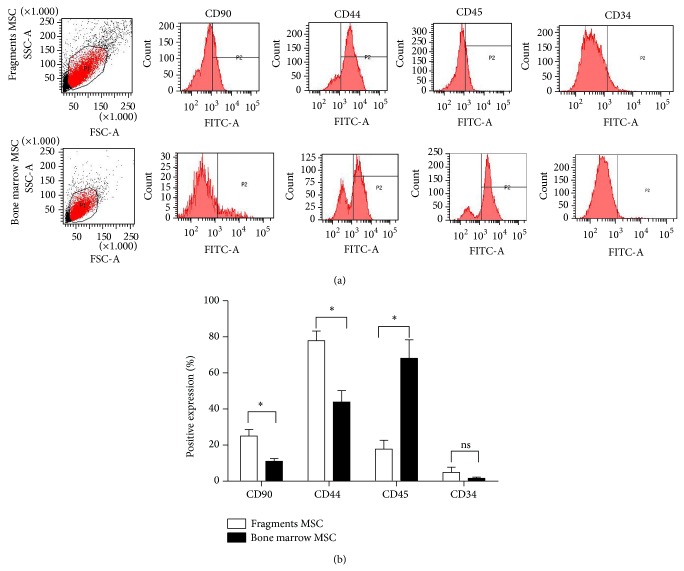
Cell immunophenotypes of the first passage from cultured bone marrow and bone fragments. (a) Representative images of CD90, CD44, CD45, and CD34 expressions. (b) Surface immunophenotype analyses. ^*^ *P* < 0.05, ns, *P* ≥ 0.05.

Homogeneous BMSCs were positive for CD90 and CD44 and were negative for CD45 and CD34 [16]. Based on the immunophenotypes with flow cytometry, we assessed the proportion of real BMSCs in cells of the first passage, which were usually contaminated by hematopoietic stem cells, macrophagocytes, and adipocytes. The cell populations expressing CD90 and CD44 from cultured fragments increased to approximately twofold the quantity of cells from cultured bone marrow, respectively (*P* < 0.05). Compared with the CD45 expression of the cells from cultured bone marrow, CD45 expression of those from cultured fragments significantly decreased (*P* < 0.05). No obvious difference was observed in the CD34 expression (Figures 4(a) and 4(b)). Data showed that more real BMSCs were obtained from cultured fragments among cells of the first passage.

### 3.4. Osteogenic Differentiation

**Figure 5:**
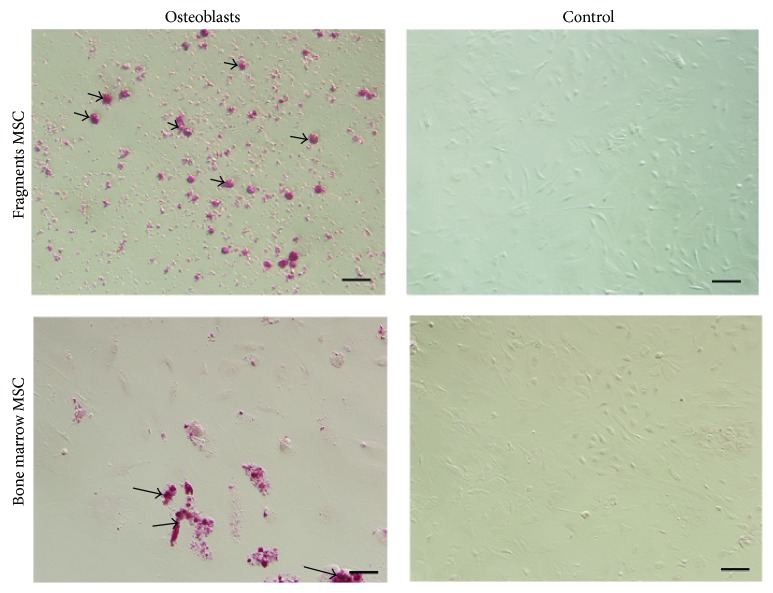
Cell osteogenic differentiation of the first passage from cultured bone marrow and bone fragments. Arrow showed the calcium nodules stained by alizarin red. Bar 50 *μ*m.

The cells were maintained in osteoinductive medium or in DMEM/F12 containing 10% FBS as the control. Some cells of the first passage revealed characteristics of osteoblasts after the osteogenic induction as assessed with alizarin red staining, whereas none of the control showed any osteogenic phenotype. Numerous calcium nodules were detected in the cells from cultured fragments. In particular, calcium nodules induced from the cells of cultured fragments seemed small and spread over the plates relatively, whereas those induced from the cells of cultured bone marrow were sporadic (Figure 5).

### 3.5. Adipogenic Differentiation

**Figure 6:**
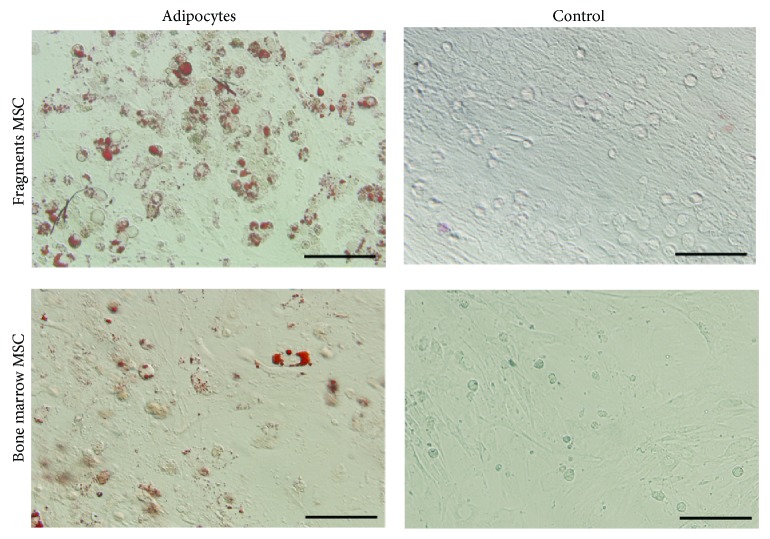
Cell adipogenic differentiation of the first passage from cultured bone marrow and bone fragments. Arrow showed the lipid droplets stained by oil red. Bar 50 *μ*m.

Although the cells of the first passage from cultured fragments and bone marrow had the specific differentiation into adipocytes, more lipid droplets were stained with oil red in cells from cultured fragments (Figure 6). Combined with the result of osteogenic differentiation, the current information indicated that cells of the first passage from cultured fragments exhibited potentially improved capacity of multilineage differentiation.

## 4. Discussion

Mouse BMSCs were successfully isolated from the adherent culture of compact bone fragments without any pretreatment of enzymes. Compared with the cells of the first passage from cultured bone marrow, cells from cultured fragments exhibited significantly enhanced proliferation and presence of more real BMSCs with potentially improved multilineage differentiation capacity.

The application of mouse BMSCs isolation by cultured bone marrow is limited by the common contamination of heterogeneous cell populations, such as hematopoietic stem cells, endothelial cells, and macrophages, and by the prolonged culture for at least several weeks, which leads to cell senescence and loss of home capacity [12, 17]. Other techniques, including immunomagnetic selection [15] and retroviral infection [18], are not standardized as common protocols easily. Short et al. [19] have reported that BMSCs reside in compact bone; this finding is then confirmed by other studies in the adherent culture of collagenase-digested fragments or the released cells after the collagenase digestion [13, 20]. The present study determined that BMSCs could be isolated from the directly adherent culture of fragments without the collagenase pretreatment, although the comparison between the culture of collagenase-free fragments and collagenase-incubated fragments was insufficient. This isolation is apparently simple and complements the assumption of BMSC source and migratory characteristic. These BMSC-like cells of the first passage from cultured fragments showed considerably high proliferation capacity. Thus, the BMSCs earlier than the tenth generation can be served as appropriate stem cell resource without obvious senescence for homotransplantation-based therapy in mouse models, which is one of the commonly used models before clinical trials. Nevertheless, isolating BMSCs from compact bone fragments is restricted to laboratory animals. The isolation is unavailable for patients in the clinical trials.

In 10-day primary inoculation, the cells from cultured bone marrow increased confluence to 80% earlier than those from cultured fragments. However, the difference of cell yields after the trypsinization between the uses of the two isolations was not statistical. Excessive and spindle-shaped cells maintained adherence to the plastic after the trypsinization in bone marrow cultivation. This finding indicated that the heterogeneity of whole bone marrow cells inhibited BMSC proliferation or the trypsinization and thereafter influenced the cell yields. Compared with the procedure that involved flushing the bone marrow out of bone cavity for 10 times, the procedure of fragments adherence to plastic for the automatic migration of BMSCs from the compact bones avoided cell viability impairment and retained proliferation potential. Moreover, the procedure that involved bone marrow elimination by flushing twice could reduce the contamination of heterogeneous cell populations and promote the colonies of BMSC.

CD90 and CD44 expressions of cells of the first passage from cultured fragments were one time higher than those of cells from cultured bone marrow, suggesting that the quantity of real BMSCs obtained from cultured fragments was improved. Unfortunately, the CD90 expression of cells by using the two isolations was still low in the primary culture. Several cell generations can increase CD90 expression and other strategies to obtain more real BMSCs in the primary culture are still needed. The high CD45 expression of cells of the first passage from cultured bone marrow was attributed to the common contamination of heterogeneous populations, particularly hematopoietic stem cells. Consistent with MSC characterization by flow cytometry, the expression changes of MSC molecular hallmarks or those that reflect cell senescence state, such as stromal-derived factor-1 and klotho, also identify MSC secretion capacity and viability and assess MSC quantity as well. Unfortunately, this information remained unknown in the current study. Some cells of the first passage showed the osteogenic and adipogenic differentiation capacity, whereas more calcium nodules in the osteoinduction and more lipid droplets in the adipoinduction of the cells from cultured fragments were observed. These data further demonstrated the presence of more real BMSCs in the primary culture of compact bone fragments and that the BMSCs maintained potentially improved multilineage differentiation capacity. Different morphology of calcium nodules induced in the culture of cells from bone fragments and whole bone marrow was also observed. Hence, detailed investigations are needed to clarify the comparisons of multilineage differentiation potential between the real BMSCs by using the two isolations in the following study.

## Figures and Tables

**Table 1:** Cell yields of the first passage from cultured bone marrow and bone fragments.

	Cells of the first passage	
	Whole bone marrow	Bone fragments
Culture (days/wells)	10/3	10/3
Cell yield (10^5^/well)	1.20 ± 0.41	0.75 ± 0.19
Cell yield (10^5^/mouse)	3.50 ± 1.23	2.25 ± 0.57

Bone marrow and fragments were cultured in 3 wells of 6-well plate from two femurs and two tibiae of a mouse for 10 days. Cells yields of cultured bone marrow were slightly higher than those of cultured fragments (*P* ≥ 0.05).
